# Supplementary material for: Rhythmic interactions between the mediodorsal thalamus and prefrontal cortex precede human visual perception
Source: Nat Commun. 2022 Jun 29;13:3736. doi: 10.1038/s41467-022-31407-z (PMC9243108; doi:10.1038/s41467-022-31407-z)
Supplement: Supplementary file 3 — Reporting Summary [file 41467_2022_31407_MOESM3_ESM.pdf]

nature portfolio

Corresponding author(s): Prof. Tobias Staudigl

Last updated by author(s): Apr 19, 2022

Reporting Summary

Nature Portfolio wishes to improve the reproducibility of the work that we publish. This form provides structure for consistency and transparency in reporting. For further information on Nature Portfolio policies, see our Editorial Policies and the Editorial Policy Checklist.

Statistics

For all statistical analyses, confirm that the following items are present in the figure legend, table legend, main text, or Methods section.

n/a	Confirmed	
☐	☒	The exact sample size (n) for each experimental group/condition, given as a discrete number and unit of measurement
☐	☒	A statement on whether measurements were taken from distinct samples or whether the same sample was measured repeatedly
☐	☒	The statistical test(s) used AND whether they are one- or two-sided<br>*Only common tests should be described solely by name; describe more complex techniques in the Methods section.*
☐	☒	A description of all covariates tested
☐	☒	A description of any assumptions or corrections, such as tests of normality and adjustment for multiple comparisons
☐	☒	A full description of the statistical parameters including central tendency (e.g. means) or other basic estimates (e.g. regression coefficient) AND variation (e.g. standard deviation) or associated estimates of uncertainty (e.g. confidence intervals)
☐	☒	For null hypothesis testing, the test statistic (e.g. F, t, r) with confidence intervals, effect sizes, degrees of freedom and P value noted<br>*Give P values as exact values whenever suitable.*
☐	☒	For Bayesian analysis, information on the choice of priors and Markov chain Monte Carlo settings
☒	☐	For hierarchical and complex designs, identification of the appropriate level for tests and full reporting of outcomes
☒	☐	Estimates of effect sizes (e.g. Cohen's d, Pearson's r), indicating how they were calculated

Our web collection on statistics for biologists contains articles on many of the points above.

Software and code

Policy information about availability of computer code

Data collection	The experiment was conducted using Presentation (Neurobehavioral Systems, Version 14.8) software.
Data analysis	The data was analysed using Matlab 2020a, the toolbox Fieldtrip (accessed in February 2020; http://fieldtrip.fcdonders.nl/), Lead-DBS (accessed in summer 2020; https://www.lead-dbs.org/) and custom code. Custom code used for this study is available at: https://github.com/StaudiglLab/corticothalamic-connect.

For manuscripts utilizing custom algorithms or software that are central to the research but not yet described in published literature, software must be made available to editors and reviewers. We strongly encourage code deposition in a community repository (e.g. GitHub). See the Nature Portfolio guidelines for submitting code & software for further information.

Data

Policy information about availability of data

All manuscripts must include a data availability statement. This statement should provide the following information, where applicable:

- Accession codes, unique identifiers, or web links for publicly available datasets
- A description of any restrictions on data availability
- For clinical datasets or third party data, please ensure that the statement adheres to our policy

Owing to additional privacy precautions relating to patient data, the associated data is available upon reasonable request.

Field-specific reporting

Please select the one below that is the best fit for your research. If you are not sure, read the appropriate sections before making your selection.

☒ Life sciences ☐ Behavioural & social sciences ☐ Ecological, evolutionary & environmental sciences

For a reference copy of the document with all sections, see nature.com/documents/nr-reporting-summary-flat.pdf

Life sciences study design

All studies must disclose on these points even when the disclosure is negative.

Sample size	Six participants were analysed. This sample size was solely driven by the supply of patients (an incredibly rare sample). Twelve additional healthy controls were included. This sample matches the sizes seen in other similar studies (see https://osf.io/tyfwu/).
Data exclusions	Artifactual trials and channels were removed from the data to ensure that the effects we observed were not driven by noise
Replication	The MEG phase bifurcation observed in the patient data was successfully replicated once in an independent test of twelve healthy controls. Replication of the thalamic signal was not possible due to rarity of these recordings.
Randomization	Not applicable - the within-participants design meant all participants took part in all conditions.
Blinding	Participants were unaware of the main hypotheses of this experiment. Participants were not allocated to groups during data collection and analysis (within-subjects design), thus no blinding of experimenters to group allocation necessary. Experimenters were unaware of whether any given trial at the moment of recording was a "hit" or a "miss", preventing any undue influence over the results.

Reporting for specific materials, systems and methods

We require information from authors about some types of materials, experimental systems and methods used in many studies. Here, indicate whether each material, system or method listed is relevant to your study. If you are not sure if a list item applies to your research, read the appropriate section before selecting a response.

Materials & experimental systems

n/a	Involved in the study	
☒	☐	Antibodies
☒	☐	Eukaryotic cell lines
☒	☐	Palaeontology and archaeology
☒	☐	Animals and other organisms
☐	☒	Human research participants
☒	☐	Clinical data
☒	☐	Dual use research of concern

Methods

n/a	Involved in the study	
☒	☐	ChIP-seq
☒	☐	Flow cytometry
☒	☐	MRI-based neuroimaging

Human research participants

Policy information about studies involving human research participants

Population characteristics	Six patients (66.6% female, mean age: 41.2 ± 8.9 years, 100% right-handed) and an additional 12 healthy controls (50% female, mean age = 27.6 ± 6.5 years, 100% right-handed) took part
Recruitment	Patient recruitment involved asking those undergoing the relevant surgery to take part following the implantation of the DBS electrodes. The healthy controls were recruited from Otto-von-Guericke University, Magdeburg, Germany.
Ethics oversight	The measurements were approved by the Ethics Commission of the Medical Faculty of the Otto-von-Guericke University, Magdeburg.

Note that full information on the approval of the study protocol must also be provided in the manuscript.
